# CP-25, a novel compound, protects against autoimmune arthritis by modulating immune mediators of inflammation and bone damage

**DOI:** 10.1038/srep26239

**Published:** 2016-05-17

**Authors:** Yan Chang, Xiaoyi Jia, Fang Wei, Chun Wang, Xiaojing Sun, Shu Xu, Xuezhi Yang, Yingjie Zhao, Jingyu Chen, Huaxun Wu, Lingling Zhang, Wei Wei

**Affiliations:** 1 Institute of Clinical Pharmacology, Anhui Medical University, Key Laboratory of Anti-inflammatory and Immune Medicine, Ministry of Education, Collaborative Innovation Center of Anti-inflammatory and Immune Medicine, Hefei, 230032, China

## Abstract

Paeoniflorin-6′-O-benzene sulfonate (code: CP-25), a novel ester derivative of paeoniflorin (Pae), was evaluated in rats with adjuvant-induced arthritis (AA) to study its potential anti-arthritic activity. AA rats were treated with CP-25 (25, 50, or 100 mg/kg) from days 17 to 29 after immunization. CP-25 effectively reduced clinical and histopathological scores compared with the AA groups. CP-25-treated rats exhibited decreases in pro-inflammatory cytokines (IL-1β, IL-6, IL-17 and TNF-α) coupled with an increase in the anti-inflammatory cytokine TGF-β1 in the serum. CP-25 treatment inhibited M1 macrophage activation and enhanced M2 macrophage activation by influencing cytokine production. Decreases in Th17-IL-17 and the Th17-associated transcription factor RAR-related orphan receptor gamma (ROR-γt) dramatically demonstrated the immunomodulatory effects of CP-25 on abnormal immune dysfunction. In addition, CP-25 suppressed the production of receptor activator of nuclear factor kappa B ligand (RANKL) and matrix metalloproteinase (MMP) 9, which supported its anti-osteoclastic effects. The data presented here demonstrated that CP-25 significantly inhibited the progression of rat AA by reducing inflammation, immunity and bone damage. The protective effects of CP-25 in AA highlight its potential as an ideal new anti-arthritic agent for human RA.

Rheumatoid arthritis (RA) is a major chronic destructive disease worldwide and is characterized by joint swelling, synovial membrane inflammation, and cartilage and bone destruction. The etiology and pathophysiology of RA are not clearly understood, and many cell types, such as fibroblasts, T cells, B cells, monocytes/macrophages and dendritic cells (DCs), have been implicated. These inflammatory cells infiltrate the synovium and are further activated to release cytokines, autoantibodies, and matrix metalloproteinase (MMP), leading to cartilage and bone destruction^1^. Pro-inflammatory cytokines produced by Th17 cells (e.g., interleukin (IL)-17) and macrophages (e.g., tumor necrosis factor-α (TNF-α), IL-1β, and IL-6) play significant roles in mediating joint inflammation^2^,^3^. These cytokines are expressed in the arthritic synovium in RA and induce the expression of receptor activator of nuclear factor kappa B ligand (RANKL), which is an essential factor for osteoclast differentiation^4^. RANKL-induced osteoclastogenesis is blocked by the decoy RANKL receptor, osteoprotegerin (OPG). OPG binds to RANKL and prevents it from interacting with RANK; thus, the local RANKL/OPG ratio determines the potency of osteoclastogenesis in the bone microenvironment^5^.

A variety of conventional anti-rheumatic drugs are available for the treatment of RA, including nonsteroidal anti-inflammatory drugs (NSAIDs) and disease-modifying antirheumatic drugs (DMARDs). Methotrexate (MTX) is the most widely used small-molecule DMARD, which remains the gold standard and the cornerstone of DMARD-based RA treatment. However, all currently used DMARDs show limited efficacy, toxicity or both. In MTX treatment both in RA patients and animal model, a dose-response relation exists, and higher MTX doses are also more prone to result in side effects^6^,^7^,^8^,^9^. Although molecularly targeted agents and biological therapies have had a major impact on the management of RA^10^,^11^, they do not yet resolve all the clinical problems due to their inconsistent efficacy and comorbidities, including increased risk of infections and tumors. Thus, newer, safer and more effective anti-inflammatory and anti-arthritic therapeutic products are being sought.

The monoterpene glucoside paeoniflorin (Pae) is one of the principal bioactive components, accounting for >90% of the total glucosides of peony (TGP), and Pae accounts for the observed *in vitro* and *in vivo* pharmacological effects of TGP. TGP has been widely used for the treatment of autoimmune disorders, including RA. The anti-inflammatory and immune-regulatory properties of TGP and Pae have been extensively confirmed in our laboratory over many years^12^,^13^,^14^,^15^,^16^,^17^,^18^,^19^,^20^,^21^,^22^. However, several studies revealed that the low bioavailability (3–4%) of Pae is mainly due to its poor absorption, which is partially caused by poor permeation, efflux via P-glycoprotein and hydrolytic degradation in intestine^23^,^24^,^25^. To improve the absorption and bioavailability of Pae, the novel compound paeoniflorin-6′-O-benzene sulfonate (code: CP-25; patent number in China: ZL201210030616.4), a new ester derivative of Pae, was synthesized, separated, purified and identified (Fig. 1A). Our studies revealed that both the oral and venous pharmacokinetic parameters (i.e., t_1/2β_, MRT, Vd and CL/F) of CP-25 were increased compared with Pae^26^. In addition, CP-25 showed superior intestinal absorption compared with Pae^27^. *In vitro* studies suggested that CP-25 regulates dendritic cell (DC) function and inhibits DC maturation via prostaglandin (PG) E2 and TNF-α signaling^28^. However, the therapeutic effects and anti-arthritic mechanism of CP-25 remain unclear compared with those of Pae and TGP.

Here, we describe the anti-arthritic activity of CP-25 in a rat adjuvant-induced arthritis (AA) model of human RA^29^. Our results show that CP-25 suppressed inflammation and bone damage primarily by modulating inflammatory mediators and immune responses, particularly Th17-IL-17. Based on our results, we suggest that CP-25 should be further evaluated for its efficacy against human RA.

## Results

### CP-25 attenuates the clinical signs of AA rats

**Figure 1:**
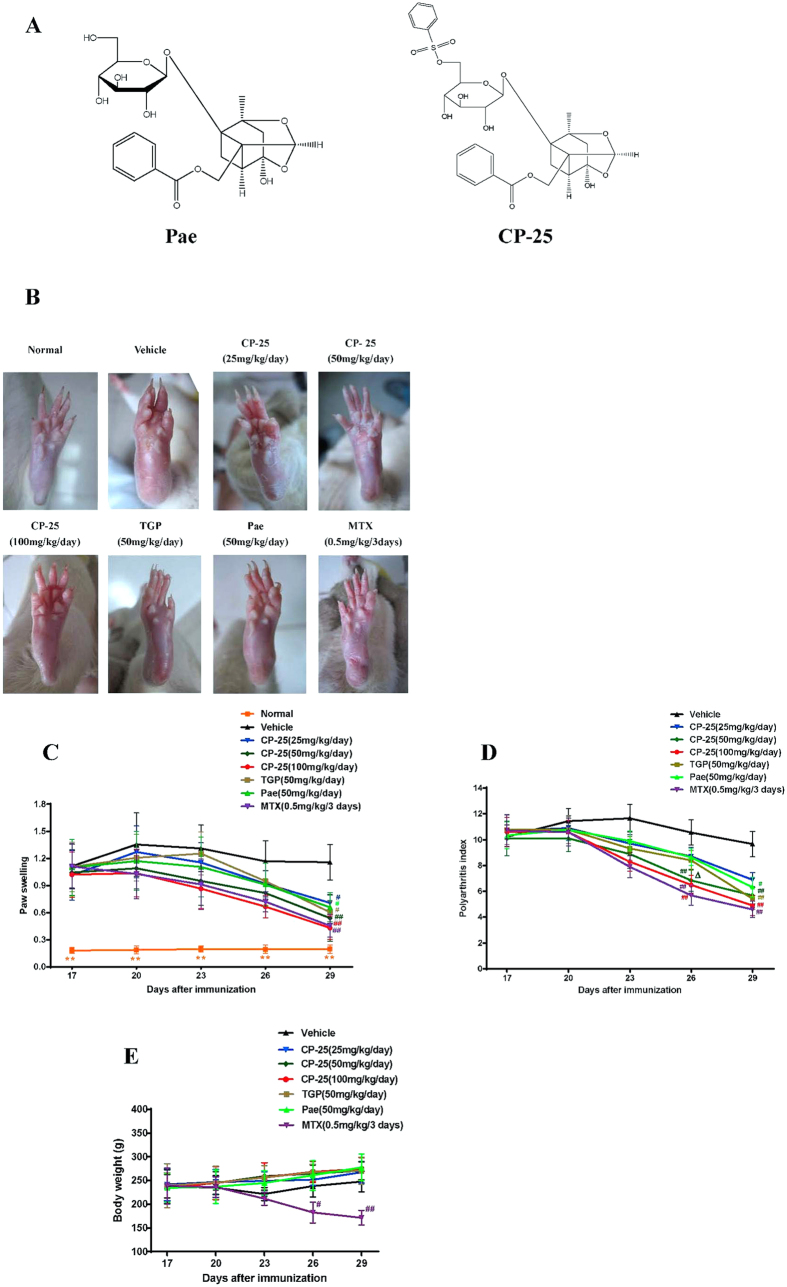
CP-25 treatment attenuates clinical signs in rats with AA. (**A**) Chemical structures of CP-25 and Pae. CP-25 (C_29_H_32_O_13_S, MW: 620); Pae (C_23_H_28_O_11_, MW: 480.45). **(B)** Photographs of representative paws from each AA group. **(C,D)** Arthritic scores of immunized Lewis rats treated either with drugs or vehicle beginning at the onset of disease (day 17) and continuing for 13 days. CP-25, TGP and Pae were administered intragastrically each day, and MTX was given intragastrically once every 3 days. Swelling of the non-injected hind paw and the polyarthritis index were assessed at 3-day intervals.^**#**^*P* < 0.05, ^**##**^*P* < 0.01 versus vehicle, ^**Δ**^*P* < 0.05 versus TGP and Pae (n = 8–10 per group). **(E**) The body weights of the Lewis rats were measured and calculated. ^**#**^*P* < 0.05, ^**##**^*P* < 0.01 versus vehicle (n = 8–10 per group).

The effect of CP-25 was evaluated using AA, a well-established *in vivo* model of inflammatory joint diseases. Arthritis developed rapidly in rats after a single injection of CFA. By days 13–16 following immunization, significant paw swelling and an increased polyarthritis index were observed in the model group compared with baseline levels, and these effects plateaued at approximately days 20–23. As shown in Fig. 1, CP-25 treatment delayed the onset of arthritis and attenuated the severity of AA in a dose-dependent manner. In comparison, CP-25, at 25 mg/kg, yielded a slight decrease in paw volume (Fig. 1C) and polyarthritis index (Fig. 1D), and excellent antirheumatic properties was observed in the groups given 50 or 100 mg/kg of CP-25. The arthritic scores in the group treated with 50 mg/kg of CP-25 were lower than those of the TGP (50 mg/kg)- and Pae (50 mg/kg)-treated rats (*P* < 0.05). In addition, we found that CP-25 administration (50 or 100 mg/kg) starting from day 26 (after AA induction), but not TGP and Pae, showed significant efficacy. Treatment with MTX (0.5 mg/kg) displayed excellent antirheumatic properties. These results showed that CP-25 inhibited the progression of arthritis in rats and possessed potent anti-arthritic activity.

The body weight of each rat was recorded every 3 days following arthritis induction (day 0). All rats gained weight at a similar rate prior to drug administration. TGP, Pae and CP-25 treated rats continued the normal increase in weight, indicating that they were well tolerated at the tested doses; whereas MTX caused a substantial reduction in body weight (Fig. 1E). Some adverse events were also detected in the rats treated with 0.5 mg/kg of MTX, including obvious signs of hair loss, loss of appetite and lack of movement.

### CP-25 improves ankle joint and spleen histopathology in arthritic rats

**Figure 2:**
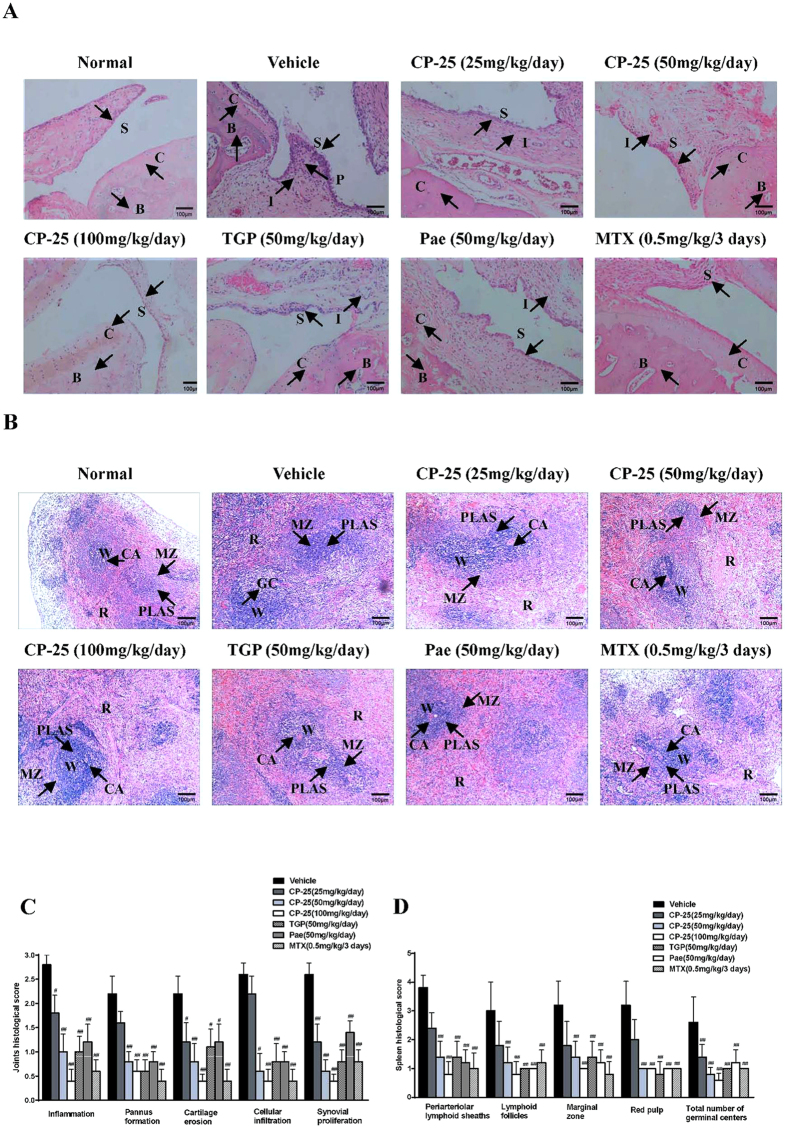
CP-25 improves arthritic joints and spleen histopathology in AA rats. Arthritic joints and spleens were harvested from AA rats on day 30 post immunization, and the inflammatory scores were assessed by H&E staining. (**A**) Representative micrographs of H&E-stained histological sections of the joints are shown. Original magnification × 100. The histology section shows the synoviocytes (S), the pannus (P), the inflammatory cells (I), the bone (B), and the cartilage (C). (**B**) Representative micrographs of H&E-stained histological sections of the spleens are shown. Original magnification × 100. The histology section shows the white pulp (W), the red pulp (R), the marginal zone (MZ), the central artery (CA), the germinal center (GC) and the periarteriolar lymphoid sheaths (PLAS). (**C**) The histological appearances of the joints were scored for the presence of synovial proliferation, cellular infiltration, pannus formation, and cartilage erosion. ^**#**^*P* < 0.05, ^**##**^*P* < 0.01 versus vehicle (n = 4 per group). (**D**) The histological appearances of the spleens were scored for the presence of cellularity of the PALS, lymphoid follicles, marginal zone, red pulp and the total number of GCs. ^**##**^*P* < 0.01 versus vehicle (n = 4 per group).

Histopathological examinations of ankle joints on day 30 revealed significant differences between CP-25-treated and AA rats. AA rats developed severe arthritis, which was characterized by marked synovial proliferation, pannus formation, inflammatory cell infiltration, and erosion of articular cartilage and bone (Fig. 2A). In comparison, CP-25 (25 and 50 mg/kg) protected the rats from bone erosion and joint destruction, and almost no bone erosion was observed in the groups that received CP-25 (100 mg/kg) and MTX (0.5 mg/kg). Specifically, 50 mg/kg CP-25 strongly inhibited cartilage erosion, cellular infiltration and synovial proliferation, whereas TGP (50 mg/kg) and Pae (50 mg/kg) had mild effects (Fig. 2C).

The spleen is the largest secondary lymphoid organ in rats and also houses the peripheral B and T cell compartment. Significant white and red pulp hyperplasia, and germinal center (GC) appearance affected the spleens of all the AA rats (Fig. 2B). In contrast, CP-25 (50 and 100 mg/kg)-treated rats exhibited only minimal hyperplasia of the white and red pulp and minimal pathological changes (Fig. 2D). The effects of TGP, Pae and MTX were equivalent to those of CP-25.

### CP-25 inhibits lymphocyte proliferation in AA rats

**Figure 3:**
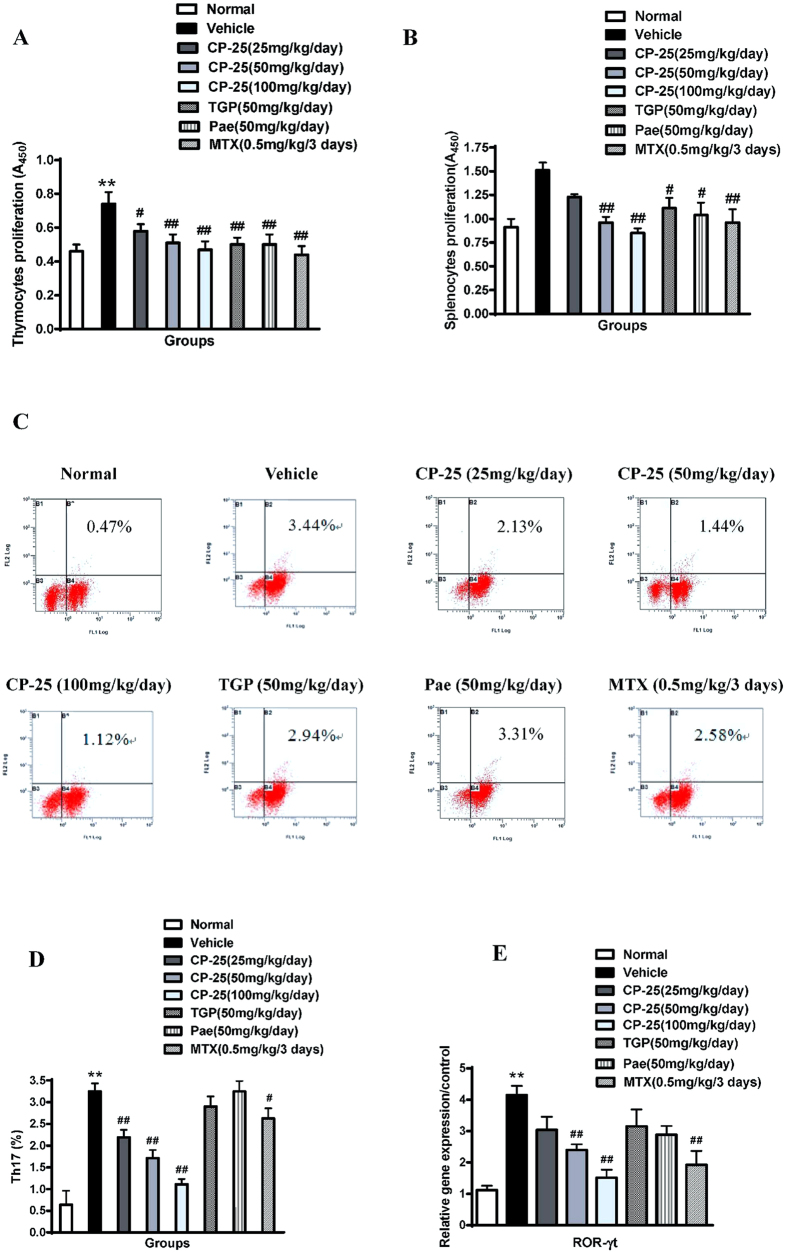
CP-25 inhibits immune activation in the rat AA model. The spleens and thymuses were harvested on day 30 after immunization (**A,B**). Splenocytes and thymocytes were suspended in 1640 medium at a concentration of 1 × 10^10^ cells/l and stimulated with LPS and Con A, respectively, for 48 h. Proliferation was examined using the CCK assay. *P < 0.05 versus normal; ^*#*^*P* < 0.05, ^#^*P* < 0.01 versus vehicle (n = 6 per group). **(C)** The Th17 cells in each group were analyzed by flow cytometry. **(D)** Bar graph illustrating the percentage of Th17 cells. **P < 0.01 versus normal; ^#^*P* < 0.05, ^##^*P* < 0.01 versus vehicle (n = 4 per group). **(E)** The ROR-γt gene expression levels relative to GAPDH were assessed in spleens via real-time quantitative PCR. **P < 0.01 versus normal; ^#^*P* < 0.05, ^##^*P* < 0.01 versus vehicle (n = 4 per group).

Inflammatory immune dysfunction supports the development of several chronic human disorders, including RA. Because B and T cells are essential for AA pathology, we asked whether the ameliorating effect of CP-25 on AA was associated with the inhibition of B and T cell effector activation. We collected the spleens and thymuses of immunized rats at the end of the study (day 30) and tested the recall responses of B and T cells *in vitro* by assessing their proliferation responses. As illustrated in Fig. 3A,B, we observed increased Con A-induced thymocyte proliferation and LPS-induced splenocyte proliferation in AA rats compared with the normal group, and CP-25 caused a concentration-dependent reduction in T- and B-cell proliferation. TGP, Pae and MTX similarly inhibited the proliferation responses. These results indicate that CP-25 regulates immune responses, at least in part, by blocking B and T cell activation.

### CP-25 decreases the percentage of Th17 cells in AA rats

Th17 cells secrete IL-17, which is thought to play a critical role in arthritis pathogenesis in RA and its animal models. To further confirm the findings that CP-25, but not TGP and Pae, specifically inhibited serum IL-17 production, we analyzed the percentage of Th17 cells in the spleen via flow cytometry. Consistent with the above findings, the percentage of Th17 cells was significantly reduced from an average of 3.44% in vehicle-treated rats to 1.12% in CP-25-treated rats (Fig. 3C,D). The transcription factor ROR-γt is vital for Th17 differentiation and IL-17 secretion. We therefore tested the effects of CP-25 on the transcription factors in the spleens of arthritic rats. Interestingly, CP-25 treatment reduced the levels of ROR-γt (Fig. 3E). This reduction might be correlated with the decreased IL-17 production and decreased Th17 cell percentage described above. TGP and Pae had no effects on Th17 cell percentages or ROR-γt expression. Thus, these results suggest that CP-25 regulates immune responses mainly by inhibiting Th17 cell activation.

### CP-25 modulates the production of macrophage-derived cytokines in AA rats

**Figure 4:**
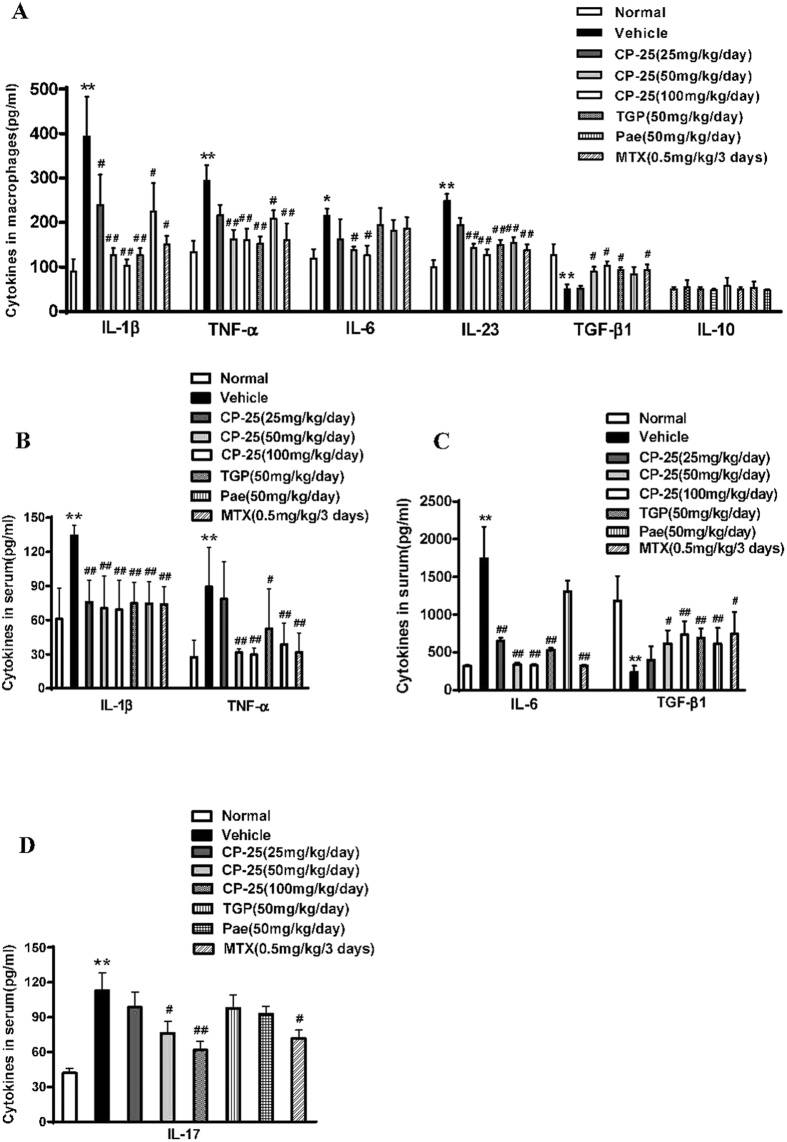
CP-25 modulates cytokines production in the serum and in macrophages in the rat AA model. Rats were sacrificed on day 30, and serum was collected from the peripheral blood. **(A)** Cytokines measured from peritoneal macrophages were also measured by ELISA. **P < 0.01 versus normal; ^#^*P* < 0.05, ^##^*P* < 0.01 versus vehicle (n = 4 per group). Serum IL-1β, TNF-α **(B)**, IL-6, TGF-β1 **(C)** and IL-17 **(D)** levels were measured via ELISA as described in the Materials and methods. **P < 0.01 versus normal; ^#^*P* < 0.05, ^##^*P* < 0.01 versus vehicle (n = 6–8 per group).

Macrophages play a central role in regulating the initiation and development of innate immune responses. To examine the effects of CP-25 on macrophage subpopulations, we measured M1-derived cytokines (e.g., TNF-α, IL-1β, IL-6 and IL-23) and M2-derived cytokines (e.g., IL-10 and TGF-β1) (Fig. 4A). As expected, macrophages from model animals expressed high levels of TNF-α, IL-1β, IL-6 and IL-23 and low levels of TGF-β1. We found that CP-25 inhibited the production of TNF-α and IL-1β and increased TGF-β1 production, similar to the effects of TGP, Pae and MTX. Regarding IL-6 levels, CP-25 (50 and 100 mg/kg) and MTX (0.5 mg/kg) yielded significant inhibition, whereas TGP (50 mg/kg) and Pae (50 mg/kg) did not. There were no significant differences between the vehicle- and drug-treated AA rats with regard to IL-10 production.

### CP-25 regulates the production of serum cytokines in AA rats

The imbalance between pro- and anti-inflammatory cytokine activities is well known to favor the induction of autoimmunity in RA. To elucidate the mechanisms underlying the improvement of AA following CP-25 treatment, we examined the serum concentrations of different immune-inflammatory cytokines in AA rats. At 50 and 100 mg/kg, CP-25 inhibited the production of proinflammatory cytokines (IL-1β and TNF-α) and increased the production of the anti-inflammatory cytokine TGF-β1, which was comparable to the effects of TGP, Pae and MTX. CP-25, TGP and MTX strongly inhibited IL-6 production, whereas Pae had no effect (Fig. 4B,C). Interestingly, CP-25 (25, 50, and 100 mg/kg) showed a strong inhibition against the production of IL-17, whereas TGP and Pae had no effect (Fig. 4D). Taken together, these results demonstrated that CP-25 inhibited the expression of critical pro-inflammatory cytokines (especially IL-17) that are related to arthritis and upregulated the anti-inflammatory cytokine TGF-β1.

### CP-25 protects against bone damage and reduces the mediators of bone remodeling and bone erosion

**Figure 5:**
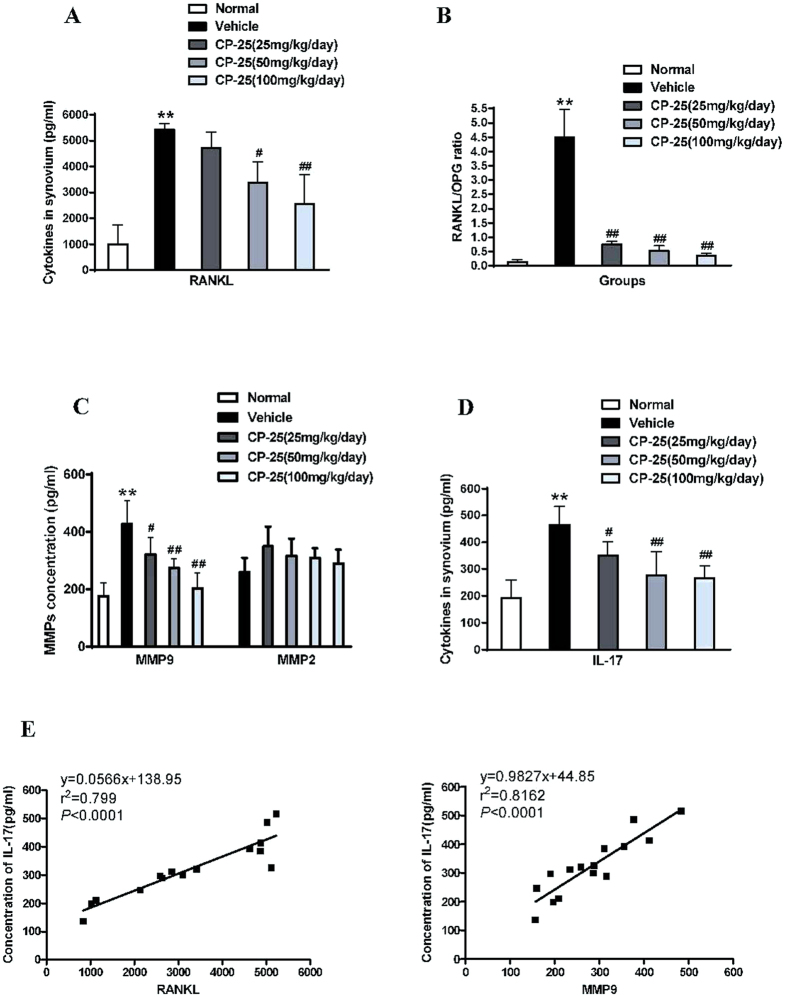
CP-25 reduces the mediators of bone destruction in the rat AA model. **(A)** RANKL, OPG, **(B)** the RANKL/OPG ratio, **(C)** MMP9, MMP2 and **(D)** IL-17. **P < 0.01 versus normal; ^**#**^*P* < 0.05, ^**##**^*P* < 0.01 versus vehicle (n = 4 per group). **(E)** Synovial IL-17 concentrations showed a significant positive correlation with the RANKL and MMP9 concentrations in the normal, vehicle and CP-25-treated groups.

To gain further insights into the mechanistic aspects of the bone damage-protective effect of CP-25, we next tested the effects of CP-25 on defined mediators of bone remodeling, e.g., RANKL and OPG, in synovial tissues. Our results revealed that CP-25 treatment inhibited RANKL production and altered the RANKL/OPG ratio in favor of anti-osteoclastogenic activity, whereas it had no significant effect on OPG (Fig. 5A,B). MMPs play critical roles in cartilage and bone destruction in arthritic joints. Therefore, we evaluated MMP2 and MMP9 production in the synovia of CP-25- and vehicle-treated rats. As illustrated in Fig. 5C, CP-25 treatment reduced MMP-9 activity in the synovium compared with normal controls. In contrast, MMP-2 production was not significantly altered in any group.

Given the specific suppression of Th17/IL-17 in AA rats, CP-25 likely regulates IL-17 production in the synovium. Consistent with the above results, CP-25 (25, 50, and 100 mg/kg) also significantly inhibited IL-17 production (Fig. 5D). Meanwhile, we found that the levels of soluble IL-17 were higher in synovial tissues than in serum from AA rats. To examine the correlation between bone damage and IL-17 levels in the inflammatory synovium, we further measured the correlation coefficients between RANKL or MMP9 levels and IL-17 levels. Interestingly, both RANKL and MMP9 (Fig. 5E) were significantly correlated with IL-17 production. Together, these results suggest that CP-25 yields a strong improvement in arthritis, likely via its unique suppression of synovial IL-17 production in autoimmune arthritis.

## Discussion

**Figure 6:**
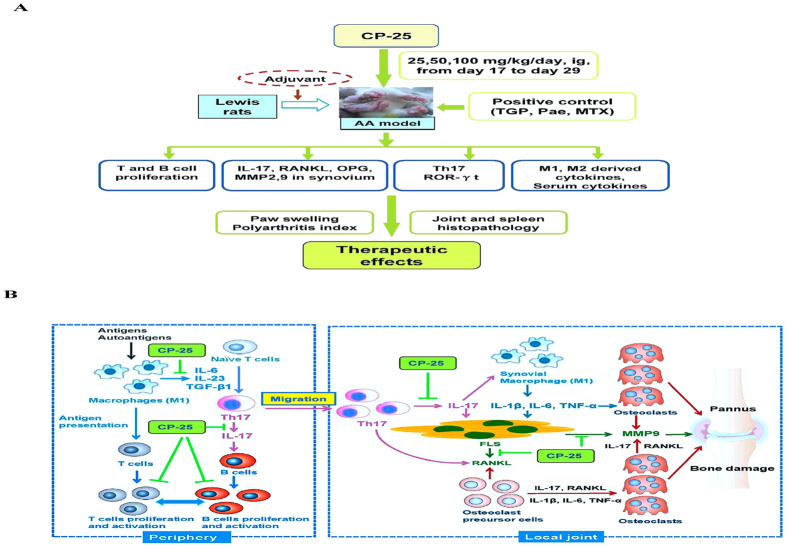
Inflammation, immune activation and bone damage in autoimmune arthritis and the effects of CP-25. (**A)** A schematic diagram showing the effects of CP-25 in AA rats. **(B)** In response to antigen/autoantigen stimulation, macrophages undergo functional polarization, which results in the generation of M1 macrophages. As important antigen-presenting cells, M1 macrophages induce the proliferation and activation of T cells. Moreover, activated M1 macrophages secrete proinflammatory cytokines such as IL-6, IL-23 and TGF-β1 and induce Th17 cell differentiation. Th17 cells secret large amounts of IL17, which also induces B cell proliferation. Unique among helper T cells subsets, Th17 cells are osteoclastogenic. Th17 cells contribute to immunity in the inflammatory phase by migrating to the inflamed joint, which is followed by Th17 cell expansion with an increase in IL-17 production. IL-17 induces the expression of RANKL by FLS and enhances the production of proinflammatory cytokines such as TNF-α, IL-1β and IL-6. IL-17 also activates synovial macrophages (M1) to secrete proinflammatory cytokines. IL-17, TNF-α, IL-1β, IL-6 and RANKL activate osteoclastogenesis and induce MMP9 production by directly acting on osteoclast precursor cells and FLS.

The current study examined the anti-arthritic activity of CP-25, which is a new ester derivative of Pae, during disease progression in a rat model of RA. The findings from this study suggest that treatment with CP-25, through reductions in immune responses, prevented bone damage by down-regulating the pro-inflammatory mediators that contribute to the progression of autoimmune arthritis (Fig. 6A).

Rat AA is a well-established *in vivo* model that has been used in numerous studies to elucidate the pathogenesis of RA and to evaluate potential therapeutic targets. The arthritic etiology of AA and RA exhibits common immunological and pathological features, including the involvement of inflammatory mediators, immune dysfunction and bone erosion^29^. Analyses of disease progression, as assessed by paw volume and the polyarthritis index, showed that CP-25 treatment markedly inhibited the development of arthritis. Histopathologic analyses of joints further demonstrated the anti-inflammatory and protective effects of CP-25 against joint damage, including cartilage and bone erosion, cellular infiltration, and synovial proliferation. Moreover, these effects were as strong as those of MTX. In this case, MTX resulted in various side effects, including obvious signs of weight loss, loss of appetite, and lack of movement, which may be due to cytotoxicity, whereas no such symptoms were detected in CP-25-treated rats, indicating that CP-25 is well tolerated at the tested doses. We suggest that CP-25 may be a potent agent for both inhibiting the progression of RA with minimal adverse effects and improving the well-being of RA patients.

The cytokine network regulates a broad range of inflammatory processes that have been implicated in the pathogenesis of RA. Pro-inflammatory cytokines (e.g., TNF-α, IL-1β, IL-6 and IL-17) mediate many of the effector responses that are associated with inflammation and bone destruction in RA^2^,^3^. The activities of these pro-inflammatory cytokines can be countered by anti-inflammatory cytokines such as TGF-β1 and IL-10^30^. To elucidate the mechanisms underlying the improvement in AA following CP-25 treatment, we determined the serum concentrations of various inflammatory mediators in AA rats. CP-25 inhibited serum TNF-α, IL-1β and IL-6 production and induced TGF-β1 production, comparable to the effects of TGP, Pae and MTX. In particular, CP-25 treatment, but not TGP and Pae, significantly reduced the levels of the pro-inflammatory Th17-cell cytokine IL-17. IL-17 is produced by Th17 cells and is known to play prominent roles in the induction and progression of arthritis^5^,^31^. We therefore examined the percentage of defined Th17 cell subsets in spleens from CP-25- and vehicle-treated arthritic rats. Our results revealed that CP-25-treated rats exhibited significantly reduced percentages of Th17 cells in the spleen. Furthermore, CP-25 treatment significantly decreased the expression of the Th17-associated transcription factor ROR-γt^32^,^33^. These findings support the role of T-cell-mediated abnormal immune responses in the development of AA and provide a partial mechanism of action of CP-25 in preventing arthritis progression through the suppression of Th17/IL-17.

Macrophages play crucial roles in the innate and adaptive immune responses, are the principal source of inflammation mediators^34^ and are pivotal in promoting inflammation and joint destruction in RA^35^. Several subsynovial macrophages in RA patients are considered sensitive biomarkers of disease severity^36^ and the response to arthritis therapy^37^. An emerging concept that defines the function of macrophages in RA is their substantial plasticity during the course of the disease. The polarization of macrophages into M1 or M2 phenotypes may vary depending on the disease activity or the anti-arthritic therapy^38^. M1 macrophages have previously been shown to play an important role in exacerbating RA^39^,^40^. Recent studies have revealed that M1 macrophages promoteTh17 polarization by secreting pro-inflammatory cytokines and thereby contribute to synovial inflammation and bone damage in RA^41^,^42^,^43^. M1 macrophages express not only IL-6 and TNF-α but also IL-23. IL-23 acts through the IL-23 receptor (IL-23 R), which is expressed on CD4^+^ T cells, to promote Th17 cell differentiation. The primary molecular mechanism of Th17 cell development is the up-regulation of the transcription factor ROR-γ^44^.

Consistent with the above findings, we also observed a high level of the M1 cytokines TNF-α, IL-6 and IL-1β and a low level of the M2 cytokine TGF-β1 in AA rats. The reduction in IL-17 in the CP-25-treatment group might have resulted from the CP-25-mediated inhibition of M1 macrophage-derived pro-inflammatory cytokines (e.g., TNF-α, IL-6 and IL-23). The results suggest that CP-25 may then down-regulate the production of IL-17 by modulating the balance between M1 and M2 macrophages, thereby modulating inflammation and immune responses and preventing bone destruction.

Bone erosion is a central feature of RA and is associated with disease severity. More importantly, bone erosion in RA begins at an early stage of the disease and is intertwined with inflammation and autoimmunity. The interaction between bone and the immune system is referred to as “osteoimmunology”^45^,^46^. RANKL is a typical osteoimmunological molecule that plays major roles in mediating the regulation of bone by immune cells^45^. How the immune system mediates bone destruction in RA has long been a challenging question. Th17 cell infiltration is a hallmark of the pathogenesis of RA, with these cells functioning as osteoclastogenic Th cells^47^,^48^. IL-17 is a potent inducer of RANKL expression in osteoblasts and FLS and induces osteoclast formation and bone resorption^49^. Moreover, IL-17 is well known to induce local inflammation and to activate synovial macrophages to secrete inflammatory cytokines such as TNF-α, IL-1β and IL-6^50^. These cytokines activate osteoclastogenesis in the inflamed synovium by either acting on osteoclast precursor cells or inducing RANKL on FLS, thereby indirectly and directly resulting in bone destruction^47^. Th17 cells also express RANKL, which might further contribute to the enhanced osteoclastogenesis (Fig. 6B). Osteoimmunological pleiotropy might explain the dramatic efficacy of biological agents that inhibit cytokines^51^ and intercellular protein kinases^52^ in ameliorating or even preventing bone destruction.

In the present study, because histological examinations of arthritic joints demonstrated that CP-25 treatment provided marked protection against inflammation and bone damage, we explored an array of mediators in the inflammatory synovium, including those associated with IL-17. Our results demonstrated that treating AA rats with CP-25 inhibited the RANKL/OPG ratio in the synovium in favor of an anti-osteoclastic effect, which was achieved primarily via reduced RANKL expression. The protective effect against bone erosion can also be explained by the reduced levels of IL-17. This supposition is further supported by the results of our *in vitro* experiments, which demonstrated that CP-25 led to reduced IL-17 signaling in the FLS of autoimmune arthritis (unpublished). CP-25 treatment significantly reduced MMP-9 production, which can be explained in part by the inhibitory effects of CP-25 on IL-6, IL-17 and RANKL, all of which are positive inducers of MMP-9^53^,^54^,^55^,^56^. Our results also highlight another interesting interaction between MMP-9 or RANKL and IL-17. Reduced RANKL and MMP-9 expression may limit the bone destruction associated with arthritis by decreasing IL-17 expression. Furthermore, the fact that CP-25 protects bone extends the spectrum of currently described immune-bone interactions and suggests dual roles in both the immune system and bone.

In conclusion, the data presented here have demonstrated that CP-25 treatment had a profound therapeutic effect on rats with AA that was consistent with reductions in immune inflammation and bone damage. Hence, these results suggest that the novel compound CP-25 represents a potentially new immunotherapeutic agent for RA, as well as for other autoimmune diseases. Further studies are required to elucidate the mechanisms involved in RA. For example, the changes in macrophage phenotypes in the different developmental stages of RA and the potential molecular mechanism by which the polarization of the M1 and M2 phenotypes is regulated require further examination. In addition, we will further explore the “osteoimmunology” mechanisms (e.g., RANKL-IL-17 cross-talk) underlying the inhibition of bone damage in CP-25-treated arthritic joints compared with the vehicle group.

## Materials and Methods

### Animals

Lewis rats (male, 150–180 g, Certificate No. LLSC2013007) were obtained from Weitong Lihua (Beijing, China) and then maintained in the institutional animal care facility. We used Lewis rats for consistency with our previous studies^57^,^58^. All experiments were approved by the Ethics Review Committee for Animal Experimentation of the Institute of Clinical Pharmacology, Anhui Medical University. All experiments were conducted according to the animal care and use committee guidelines.

### Reagents

TGP (>40% purity) and Pae (>90% purity) were provided by the Chemistry Laboratory of the Institute of Clinical Pharmacology of Anhui Medical University (Hefei, Anhui Province, China). MTX was purchased from Shanghai Pharmaceutical (Group) Co., Ltd. (Shanghai, China). Lipopolysaccharides (LPS) were purchased from Sigma Chemical Co. (St. Louis, USA). Concanavalin A (Con A) was purchased from Biosharp Co. (USA). Cell counting kit-8 (CCK-8) was purchased from Dojindo Laboratories (Japan). Enzyme-linked immunosorbent assay (ELISA) kits for TNF-α, IL-1β, IL-6, IL-10, IL-23, TGF-β1, MMP2 and MMP9 were purchased from R&D Systems. ELISA kits for RANKL and OPG were purchased from Cusabio BioTech Co., Ltd. CD4-FITC and IL-17-PE antibodies were purchased from Biolegend Co. (USA).

### CP-25

Pae (3 g) and dimethylamino-pyridine (DMAP) (75 mg) were dissolved in a solvent composed of a mixture of chloroform and pyridine (16 ml + 6 ml). An appropriate benzenesulfonyl chloride was added to start the reaction. The mixture was stirred at room temperature for 8 h until the nearly complete conversion of Pae was achieved. Reaction progress was monitored by TLC on a GF254 (Hai Yang Chemical Factory, Qing Dao, Shandong, China), and column chromatography was performed to purify the final product with an eluent consisting of dichloromethane/methanol (30/1, v/v). The purity of the white powder was 98.8%, as determined by HPLC.

### Induction of AA

AA was initiated in the Lewis rats via intradermal immunization in the left hind metatarsal footpad with 100 μl (1 mg/rat) of heat-killed *Mycobacterium butyricum* in liquid paraffin (Complete Freund’s adjuvant). The day of the first immunization was defined as day 0. On days 13–16 after the administration of the adjuvant, when joint inflammation in all the rats reached a maximum in the experiment, the animals were randomly divided into groups (n = 10 per group) such that there were no significant differences between the groups.

### Treatment of AA

Adjuvant-injected rats were divided into seven groups, in which AA rats were intragastrically administered CP-25 (25, 50, or 100 mg/kg/day), TGP (50 mg/kg/day), or Pae (50 mg/kg/day) and MTX (0.5 mg/kg, every 3 days) from days 17–29 after immunization. CP-25, TGP, Pae and MTX were suspended in 0.5% sodium carboxymethylcellulose (CMC-Na) before use. In the normal and AA model groups, the rats were given an equal volume of vehicle (CMC-Na) at the same time.

### Clinical assessment of AA

AA severity was evaluated by a single observer who was blinded to the treatment conditions. From day 7 after immunization, the rats were examined every 2 days for paw volume and polyarthritis index. Footpad volume was measured with a water replacement plethysmometer. Arthritis sores were assessed as detailed previously^57^,^59^. The polyarthritis index was monitored using a macroscopic scoring system ranging from 0 to 4 per limb, yielding a total score of 0 to 16 per animal.

### Histological examination

The rats were anaesthetized and sacrificed on day 30 after immunization. The secondary ankle joints and spleens were removed, fixed in 10% neutral-buffered formalin, and then decalcified in 5% formic acid and embedded in paraffin. The sections (5 mm) were stained with hematoxylin and eosin (HE) and examined microscopically as described previously^57^. Briefly, the ankle joints were histopathologically analyzed for inflammation, synovial proliferation, cellular infiltration, pannus formation, and cartilage erosion (scales 0–4). To assess the extent of spleen remodeling, histological sections of rat spleens were scored (scale, 0 to 4). The evaluation parameters were cellularity of periarteriolar lymphoid sheaths (PALS), lymphoid follicles, marginal zone, red pulp and the total number of GC. The pathological evaluations were performed randomly by a pathologist who was blinded to the groups of the specimens.

### Thymocyte and splenocyte proliferation assay

The thymus and spleen were harvested at day 30 after immunization. Thymocytes and splenocytes were isolated using routine methods. These cells were suspended in RPMI-1640 (HyClone, Carlsbad, CA, USA) medium at a concentration of 1 × 10^10^ cell/l. Thymocytes (100 μl) and 100 μl Con A (at a final concentration of 5 mg/l) or splenocytes (100 μl) and 100 μl LPS (at a final concentration of 4 mg/l) were added into 96-well flat-bottomed culture plates. The cultures were incubated at 37 °C in 5% CO_2_ for 48 h. Four hours before the termination of the culture, 10 μl of CCK-8 (Dojindo Laboratories, Kumamoto, Japan) was added to each well. After incubation at 37 °C for an additional 4 h, the absorbance (A) was measured using an EJ301 ELISA Microwell Reader at 450 nm.

### Macrophage isolation

Peritoneal macrophages from the rats were prepared via intra-abdominal injection of cold PBS. After several minutes, the peritoneal cells were collected and washed. The cell suspensions were adjusted to 2 × 10^9^ cell/l in RPMI-1640 containing 10% FCS and dispensed at 1 ml/well into 24-well plates. After incubation for 2 h at 37 °C in a humidified 5% CO_2_ atmosphere, the nonadherent cells were removed by washing twice with RPMI-1640. Next, 100 μl of LPS (at a final concentration of 4 mg/l) plus 0.9 ml RPMI-1640 medium was added at 37 °C, and the plates were cultured for 48 h. The cultures were centrifuged (1000 × *g*, 5 min), and the sample supernatants were collected and stored at −80 °C until use.

### Measurement of the mediators in the synovium

Synovium samples were cut into small blocks and cultured for 24 h in 2 ml of serum-free RPMI-1640 containing 0.2% lactalbumin hydrolysate in a 5% CO_2_ incubator as described previously^58^. The tissue samples were homogenized on ice in 50 mM Tris-HCl buffer, pH 7.5. The homogenates were centrifuged at 4 °C for 20 min at 10,000 × *g*. The concentrations of IL-17, RANKL, OPG, MMP2 and MMP9 in the supernatants were measured using ELISA kits.

### Cytokine measurements

The concentrations of TNF-α, IL-1β, IL-6, IL-10, IL-17, IL-23, and TGF-β1 were measured using ELISA kits according to the manufacturers’ instructions.

### ROR-γt quantification via real-time PCR analysis

Total RNA was isolated from the spleens of AA rats using Trizol reagent (Invitrogen Corp.) and then reverse transcribed using the First Strand complementary DNA (cDNA) Synthesis Kit (Invitrogen). Standardization was performed with GAPDH as the internal control. Quantitative real-time PCR (RT-PCR) was performed with the SYBR green two-step quantitative RT-PCR kit with ROX (Invitrogen) according to the manufacturer’s manual and as previously described^60^. The following specific primers for ROR-γt were used: forward (F), 5′-CGCCTGGAGGACCTTCTACG-3′; and reverse (R) 5′-ACAGCTCCATGAAGCCTGAG-3′.

### Flow cytometry

Suspensions of single splenic cells (1 × 10^6^/ml) were stained with fluorescence-conjugated monoclonal antibodies to CD4 for 30 min at 4 °C. For intracellular staining, the cells were stimulated for 5 h with PMA, ionomycin (Sigma-Aldrich), and GolgiStop (BD) and permeabilized and stained using anti-IL-17. Cell-associated fluorescence was analyzed using a FAC scan instrument (FC500, Beckman Coulter) and the affiliated Cell Quest software.

### Statistical analysis

The results are presented as the means ± SEM. Data were analyzed by one-way ANOVA. Comparisons between two groups were performed using the Dunnett’s multiple comparisons test or post-hoc analysis. Statistical analyses were carried out using GraphPad Prism version 6.0 (GraphPad Software, San Diego, CA, USA). *P* < 0.05 was considered statistically significant.

## Additional Information

**How to cite this article**: Chang, Y. *et al*. CP-25, a novel compound, protects against autoimmune arthritis by modulating immune mediators of inflammation and bone damage. *Sci. Rep*. **6**, 26239; doi: 10.1038/srep26239 (2016).
